# Genome Characterisation of Esocid Herpesvirus 1 (EsHV-1)

**DOI:** 10.3390/v17101361

**Published:** 2025-10-11

**Authors:** Mikael Leijon, Petter Tibblin, Tobias Lilja, Fereshteh Banihashem, Björn David Persson

**Affiliations:** 1 Swedish Veterinary Agency, 751 89 Uppsala, Swedendavid.persson@sva.se (B.D.P.); 2 Ecology and Evolution in Microbial Model Systems, EEMiS, Department of Biology and Environmental Science, Linnaeus University, 391 82 Kalmar, Sweden

**Keywords:** esocid herpesvirus 1, pike, *Esox lucius*, blue spot disease, Alloherpesviridae, genomic characterization

## Abstract

The alloherpesvirus esocid herpesvirus 1 (EsHV-1) causes epidermal hyperplasia on the skin and fins of northern pike (*Esox lucius*). For the first time, we present a near-complete genome sequence of EsHV-1, directly obtained from a pike skin sample. The 223,553 bp sequence of the genome has a GC-content of 56.47% and is organised into a long, unique segment (148,159 bp) and a short, unique segment (45,925 bp). The short segment is flanked by inverted repeat sequences (IRSs) of 14,733/6 bp, with the IRS length difference attributed to a codon deletion. The genome is predicted to contain 144 open reading frames, including eight duplicated within the IRSs. The leftmost third of the genome contains genes of unknown function, but many of which exhibit extensive inter-gene homology, suggesting gene duplication. Six paralogous groups were identified, each containing two to thirteen gene members. Homologues of all twelve alloherpesvirus core genes are present. The ATPase subunit of the terminase and the DNA polymerase is composed of three and two exons, respectively. However, an alternate splicing pattern is found, for which, speculatively, a role is suggested in the terminase assembly at the capsid portal.

## 1. Introduction

The northern pike (*Esox lucius*) is a widely distributed apex predator inhabiting freshwater and brackish ecosystems across the Northern Hemisphere [1]. In the Baltic Sea, particularly in coastal areas, pikes serve as an essential top predator, filling the ecological niche left by declining cod (*Gadus morhua*) populations. By regulating mesopredatory fish such as sticklebacks (*Gasterosteus aculeatus*), pike contributes to ecosystem stability [2]. A decline in pike populations leads to an increase in mesopredatory fish, which in turn reduces populations of small invertebrates that feed on algae. This trophic shift promotes excessive phytoplankton growth, eutrophication, and harmful algal blooms, demonstrating the cascading effects of predator loss on aquatic ecosystems. The preservation of top-down control is therefore crucial for maintaining ecological stability in the Baltic Sea [3,4]. Conservation efforts, including the restoration of breeding habitats [5], enforcement of sustainable fisheries management, and protection of critical habitats, are necessary to aid the recovery of pike populations and to mitigate the trophic shift. However, pike populations face additional challenges due to their susceptibility to various viral pathogens, both RNA and DNA viruses, which may significantly impact fish health and population dynamics, but this aspect has been less studied [6].

*Alloherpesviridae* is a family within the Herpesvirales order whose species members infect fish and frogs [7]. To date 13 alloherpesvirus species have been fully sequenced [7]. Esocid herpesvirus 1 (EsHV-1) is the only alloherpesvirus known to infect pike and is associated with epidermal hyperplasia characterised by raised, bluish-white lesions on the skin and fins [8,9,10,11]. The virus has not yet been successfully propagated in vitro and has only been partly genetically characterised [8,10]. These studies indicate distant phylogenetic similarities with other fish herpesviruses, including salmovirus salmonidallo1 (SalHV-1) and ictavirus acipenseridallo2 (AciHV-2). Partial sequencing of the EsHV-1 genome, which included the DNA polymerase and terminase [8,10], demonstrated moderate sequence identity to related Alloherpesviridae members, underscoring its evolutionary divergence within this viral family [12]. Here we present a near-complete genome characterisation of an EsHV-1 and carry out a detailed analysis of the genome.

## 2. Materials and Methods

Information regarding sample collection and DNA extraction has been published [10]. For the reader’s convenience the procedures for DNA sequencing and bioinformatics are repeated here.

### 2.1. DNA Sequencing

For Illumina short-read sequencing, DNA libraries were prepared using the NEXTERA-XT kit (Illumina Inc., San Diego, CA, USA) following the manufacturer’s protocol. Library quality was verified using the Agilent 2100 Bioanalyzer (Agilent Technologies, Santa Clara, CA, USA). Libraries were then sequenced on an Illumina MiSeq instrument (Illumina Inc., San Diego, CA, USA) using a MiSeq Reagent Kit v3 in a 2 × 300-cycle paired-end run. For MinION long-read sequencing, the DNA concentration was measured using a Qubit broad-range dsDNA assay (Invitrogen, Waltham, MA, USA). A 1 μg sample was used to prepare the sequence library with the Ligation Sequencing Kit SQK-LSK110 (Oxford Nanopore, Oxford, UK), following the standard protocol. The sample was sequenced on a MinION flow cell 9.4 for 72 h, generating approximately 336 k reads (265 megabases) with a pass quality filter (Q10) using super-accurate base calling in Minknow (Oxford Nanopore, Oxford, UK).

### 2.2. Bioinformatics

Paired-end short reads were initially processed for quality using Trimmomatic v. 0.39 [13]. The trimming criteria included a sliding window of four nucleotides and a required average quality score of 15. Following trimming, both the resulting short reads and the MinION long reads were subjected to hybrid de novo assembly using SPAdes v. 3.15.4 [14] in standard mode. The assembled contigs were classified using DIAMOND v. 2.0.9 [15] with a database constructed from the NCBI nr database (GenBank release 248) and the corresponding NCBI taxonomy databases. The final assembled genome was annotated using PROKKA v. 1.14.5 [16]. To identify potential introns, further annotations were conducted with HMMGene v. 1.1 [17]. However, the terminase gene was annotated manually, consistent with published results (e.g., WWU01670). Tentative protein assignments for the coding sequences (CDS) were performed using the NCBI BLASTp online service https://blast.ncbi.nlm.nih.gov/Blast.cgi (accessed on 1 July 2025). Paralogue genes were searched by using BLASTx with the EsHV-1 genome as a query and the investigated genes as a target with an e-value cut-off of 0.001.

To identify large-scale repeat regions, dot plots were generated. A window size of 9 nucleotides and a similarity cut-off level of 70% was used in the analysis, with any repeating sequences appearing as off-diagonal lines.

Phylogenetic analysis was performed on 12 concatenated alloherpesvirus core proteins. The alloherpesvirus core proteins include the DNA polymerase catalytic subunit, helicase-primase subunits (helicase and primase), DNA packaging terminase subunit 1, major capsid protein, capsid triplex subunit 2, and capsid maturation protein, along with five Cyvirus cyprinidallo3-numbered homologs (Allo64, Allo54, Allo60, Allo37, and Allo56), which are proteins of unknown function [18]. A second tree was constructed excluding the polymerase and terminase. Each core protein was individually aligned before concatenation. Neighbour-joining phylogeny was then calculated using the Jukes-Cantor distance measure and validated with 1000 bootstrap replicates. The trees were constructed from sequences obtained from the following GenBank accessions: MK260013.1, KT387800.1, NC_019491.1, OM936983.1, OR001786.1, OK485036.1, PQ564448.1, DQ665917.1, NC_008210.1, PQ863777.1, PP622675.1, PP098466.1, MH048901.1, MG271984.1, M75136.2, PV991061.1 and OK337613.1.

Dot plots, phylogeny and genome views were created using CLC Genomics Workbench v. 24.0.1 (QIAGEN, Aarhus, Denmark).

## 3. Results

**Figure 1:**
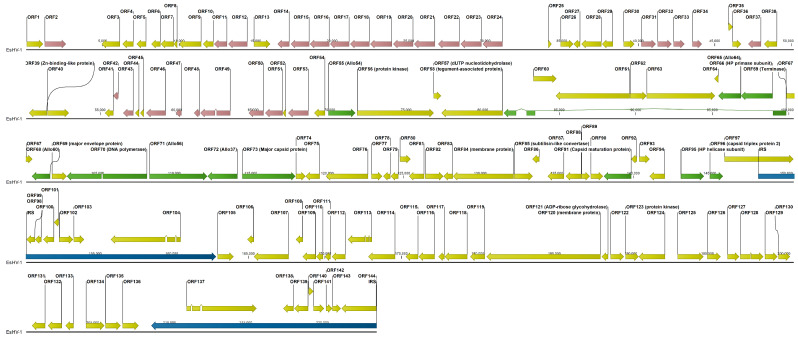
Whole genome view of EsHV-1. ORFs corresponding to the twelve core genes are shown in green. ORFs belonging to identified paralogous groups are shown in red. All other ORFs are coloured yellow. Inverted repeat sequences (IRSs) are indicated by blue arrows. The putative function of individual ORFs, where suggested by sequence homology, is labelled adjacent to each ORF.

The Illumina MiSeq and MinION sequence read data were obtained from nucleic acids extracted from a pike skin sample as previously described [10]. Hybrid assembly with SPAdes [14] yielded a 160,572 bp contig. Through detailed manual examination of overlapping MinION reads, the sequence was further extended to a length of 223,553 bp (Figure 1). The genome (GenBank accession PV991061.1) has a GC content of 56.47% and is organised into a unique long segment (UL) of 148,159 bp and a unique short segment (US) of 45,925 bp. The short segment is flanked by inverted repeat sequences (IRSs) of 14,733/6 bp, with the IRS length differing by a codon. The independent determination of the right terminal IRS using MinION reads initially showed several differences compared to the right terminal IRS. Strikingly, most of these discrepancies coincided with regions of low coverage (<10). Given the higher error rate of MinION sequencing, these low-coverage differences were attributed to sequencing errors. Therefore, the internal IRS, determined from MiSeq read data, was used to correct these discrepant regions in the right terminal IRS, as IRSs are expected to be virtually identical. Even after these corrections, differences persisted in ORF137 relative to ORF104, specifically three non-synonymous mutations, one synonymous mutation, and a codon deletion.

The genome falls into three sections of similar length. First, the left half of UL, which, with the single exception of ORF40, a Zn-binding-like protein, contains ORFs of unknown function. Many ORFs of this section display extensive, although relatively low, amino acid identity, indicating gene duplication. Six groups of paralogous ORFs can be identified. The largest group has thirteen members, ORF11–12/ORF14–23, with pairwise amino acid identity in the range 7.4–40.0%. A general trend is that nearby ORFs are more likely to have sequence identity in the higher range. Further, there are two groups: ORF46/ORF49–51 and ORF53/ORF31–34, with five and four members and amino acid sequence identities in the ranges 9.2–18.2% and 11.1–27.7%, respectively. Finally, there are three paralogous ORF pairs, ORF2/ORF37, ORF42–43, and ORF47–48, with amino acid sequence identities of 19.1%, 14.8%, and 50.8%, respectively. The ORF2/ORF37 pair is the only exception to the pattern that paralogous genes are closely located in the genome and in the same orientation (Figure 1). Thus, in total 28 ORFs are members of paralogue families in this section of the genome, while we find no paralogues in the rest of the genome. The central third of the genome carries the core genes and most other ORFs to which a function can be assigned from sequence homology. The last third of the genome is composed of the IRSs and the US. In total 144 ORFs are predicted, and eight of them are duplicated in the IRSs except for the previously mentioned differences observed between ORF104 and ORF137 and a 756 amino acid truncation of ORF144 relative to ORF97 (Figure 1). This indicates that the genome may not have been fully sequenced at the right end, which in turn implies that the IRSs could in fact be longer. Since ORF97 is closely preceded by ORF96, which is the capsid triplex protein 2, and unlikely to be part of the IRS, an upper limit for the length of the IRSs is 17,034 bp.

**Figure 2:**
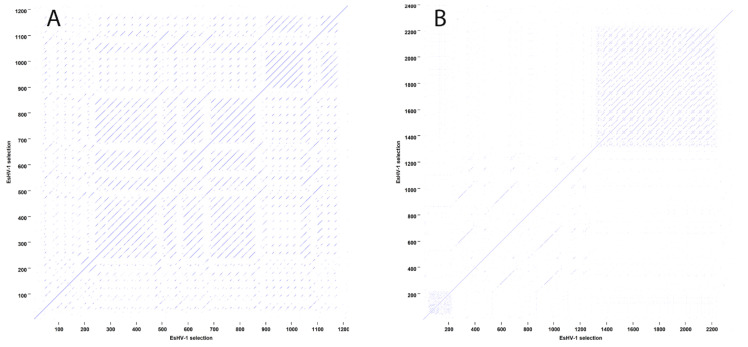
Dot plot showing the region between ORF1 and ORF2 (**A**) and between ORF40 and ORF41 (**B**). The similarity cutoff level is set to 70%.

Outside of the IRSs, there are five longer non-coding regions ranging from 1200 to 2098 bp. These regions are located between ORF2–3, ORF24–25, ORF34–35, ORF40–41, and ORF49–50 (Figure 1). For ORF24–25, BLASTx analysis indicates that the region contains two sequence segments of inactivated genes belonging to the preceding family of 13 duplicated genes. Similarly, the regions ORF34–35 and ORF49–50 contain one segment each of an inactivated duplicated gene belonging to the respective neighbouring group of duplicated genes. On the other hand, the regions ORF2–3 and ORF40–41 contain extensive sequence repeats (Figure 2). Sequence repeats are also found in several other locations: in IRSs (Figure S1); at the beginning of the genome within the coding region of ORF1; in ORF38 and its following non-coding region; and in ORF63, which is situated in the long intron of the ATPase subunit of the terminase (Figure S2).

**Figure 3:**
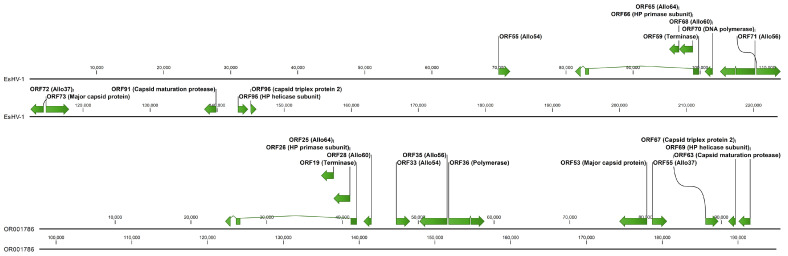
Comparison of the core genomes, shown in green, of esocid herpesvirus 1 (top) and white sturgeon herpesvirus 1 (bottom).

In Figure 3 the core genome of EsHV-1 is compared to that of the related alloherpesvirus white sturgeon herpesvirus 1 (WSHV-1), which has a similar overall genomic architecture [19]. It is seen that the core genes are situated in the middle of the genome for EsHV-1, as previously mentioned, while they are found in the first half of the genome for WSHV-1. The ATPase subunit gene is in the same orientation and composed of three exons for both viruses, with the Allo64 and primase subunit of the helicase-primase positioned right at the right end of the long intron. Next follows Allo60 in both genomes. However, while Allo60 is followed by Allo54 in WSHV-1, this gene is reversed and positioned far to the left in the EsHV-1 genome, preceding the terminase by about 10,000 bp (Figure 3). The two following genes, the DNA polymerase and Allo56, are positioned back-to-back (i.e., coding sequence on opposite strands) in both viruses, but in opposite orientations. Similarly, the major capsid protein gene and Allo37, which follows next, are also positioned back-to-back and also in opposite orientations in the two viruses. Finally, the last three core genes, capsid maturation protease, the helicase subunit of the helicase-primase, and the triplex protein 2, follow in EsHV-1. The same tree genes but all in opposite orientations and, additionally, with the helicase-primase and the triplex protein 2 genes in opposite order, end the sequence of core genes in WSHV-1 (Figure 3).

**Figure 4:**
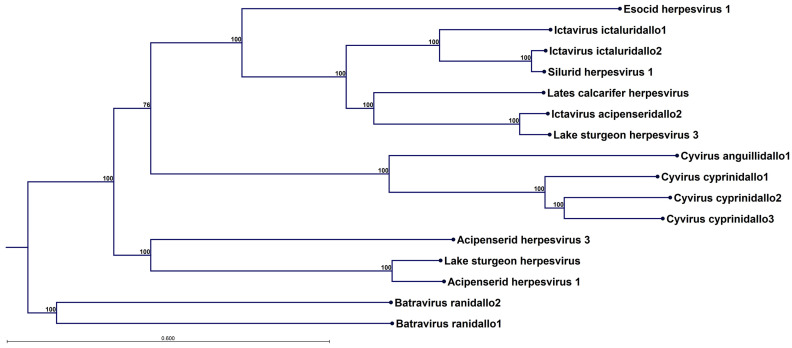
Neighbour-joining tree constructed from concatenated alignments of the 12 alloherpesvirus core proteins: DNA polymerase catalytic subunit, DNA packaging terminase subunit 1, helicase-primase subunits (helicase and primase), major capsid protein, capsid triplex subunit 2, capsid maturation protein, and the Allo37, Allo54, Allo56, Allo60, and Allo64 proteins. The bootstrap support, from 1000 replicates, is shown at the nodes. The bar signifies the branch length corresponding to the given number of substitutions per site. The tree is rooted just by the assumption that frog and fish alloherpesviruses have a common origin.

In Figure 4, a phylogenetic tree constructed from the concatenated alignment all 12 alloherpesvirus core protein for 16 different alloherpesvirus species is shown. Of the four currently defined alloherpesvirus genera—*Ictavirus*, *Cyvirus*, *Salmovirus* and *Batravirus*—three are represented, as well as a clade with unclassified viruses including acipenserid herpesvirus 1 and 3. No *Salmovirus* could be included since a complete set of core proteins is not available for this genus (the genome sequence is available at GenBank accession in OK337613, but the splice patterns have not been delineated and annotated).

However, in Figure S3, the phylogenetic tree, including salmovirus salmonidallo1, is shown for ten alloherpesvirus core proteins, excluding the polymerase and terminase. EsHV-1, although highly divergent, forms a clade together with the viruses in the *Ictavirus* genus (Figure 4) and the *Salmovirus* (Figure S3). Overall, the trees have very strong bootstrap support, but it is evident that some uncertainty is introduced by including the two proteins produced by spliced genes (cf. Figure 4 and Figure S3). It is not possible to construct an outgroup due to dissimilarity between alloherpesvirus and other viruses, but the trees have tentatively been rooted by assuming the fish and frog alloherpesviruses share a common origin.

**Figure 5:**
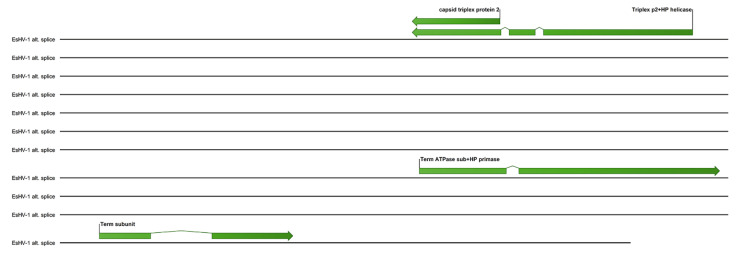
Alternate splicing scheme involving several core genes.

A possible alternate splicing scheme for the terminase and several other core genes is shown in Figure 5. Here the helicase-primase helicase subunit is spliced together with the capsid triplex protein 2, while one exon of the ATPase subunit of terminase is spliced together with the helicase-primase primase subunit. The association of the helicase-primase will then bring one exon of the terminase to the capsid triplex proteins, which line the capsid portal. The remaining two terminase exons can, in this scheme, be spliced together and associate with the third exon and thus assemble the terminase at the portal.

## 4. Discussion

Herpesviruses are large and complex viruses, often with long repeating sequences, extensive gene duplication, and genes with introns, which make sequencing and annotation challenging. For alloherpesvirus this situation is further complicated by high sequence diversity between species [12] and that up until early 2025 only 13 complete alloherpesvirus genomes have been determined [7]. Here, for the first time, a near-complete genome of the alloherpesvirus, EsHV-1, is presented. Only short genomic regions of this virus have previously been reported [8,10]. Furthermore, the genome was obtained directly from a northern pike skin sample, avoiding culturing, which may lead to alterations of the genome [20,21]. The genome organisation is the same as for SalHV-1 [22] (c.f. GenBank accession OK337613) and white sturgeon herpesvirus [19] with a long unique segment (UL) and a short unique segment (US), where the latter is flanked by inverted repeat sequences. In Figure 1 it is seen that ORF97 crosses the IRS interface to the UL with about half the ORF on either side. Correspondingly, ORF144 of the terminal IRS, otherwise identical to ORF97, is 756 codons shorter. The right terminal of the genome was independently determined using MinION long reads. However, limited coverage in combination with the higher error rate probably precluded complete determination of this end. If it is assumed that ORF144 is identical to ORF97, then the total length of the genome will be 225,821 bp.

In a recent review [7], this genome architecture is denoted “Arch-1”, which is also the most common genomic architecture among alpha herpesviruses [7]. Although in this review only Arch-2, Arch-4 and Arch-5 are listed to occur among alloherpesviruses, we thus note that Arch-1 is also prevalent and is found in SalHV-1, EsHV-1 and WSHV-1. The EsHV-1 genome is predicted to code for 144 proteins, including all 12 core proteins (Figure 1). The similarity of EsHV-1 to SalHV-1 extends beyond the overall genome architecture, and, using BLASTp analysis, it is found that the following groups of ORFs, all coding for proteins with unknown function, in EsHV-1: 60, 61, and 67; 75, 76, and 77; and 87, 88, and 93 are similar to the following groups of ORFs in SalHV-1 (GenBank accession OK337613): 79, 78, and 71; 40, 43, and 44; and 33, 32, and 26, respectively. Allowing for a potential reversal of the genome fragments, the ORFs in the two sets of groups are similarly positioned along respective genomes. For example, reversal of the SalHV-1 gene fragment carrying ORFs <71, 78, 79> would create the gene order <79, 78, 71>, which would align with ORFs <60, 61, 67> of EsHV-1. This also holds for the SalHV-1 fragment carrying the genes <26, 32, 33>, which, after reversal <33, 32, 26>, aligns with EsHV-1 ORFs <87, 88, 93>. Taken together with the comparison of the core genomes of EsHV-1 and WSHV-1 presented in the results section (Figure 3), these observations strongly suggest that recombination plays a major role in alloherpesvirus evolution. Furthermore, phylogenetic analysis confirms the divergent nature of the EsHV-1 genome and that it is most closely related to the *Ictavirus* (Figure 4) and *Salmovirus* genus (Figure S3).

An outstanding feature is that the first half of the UL segment of the EsHV-1 genome contains mainly genes of unknown function and is apparently subjected to duplication. At least 28 genes are members of six different paralogue families (Figure 1). Paralogue gene families have also been found among Cyprinid herpesviruses [18]. Inactivated paralogues also explain three out of five longer (>1200 bp) non-coding regions (Figure 1), while the remaining two such regions contain extensive repeat regions (Figure 2).

Finally, a possible alternate splicing pattern is noted (Figure 5) where the helicase-primase helicase subunit gene is spliced together with the capsid triplex protein 2 gene, while one exon of the ATPase subunit of terminase is spliced together with the helicase-primase primase subunit gene. Non-covalent affinities forming the helicase-primase complex and associating the remaining two spliced exons of the terminase to the one connected to the triplex protein 2 will then assemble the terminase at the portal, which is lined by triplex protein 2. Although this scheme is highly speculative, it is interesting to note that capsid-immobilised helicase complexes have been shown to act as molecular motors pumping single-stranded DNA into the adeno-associated virus type 2 capsid [23].

Despite the significant expansion of genomic information for microorganisms, a limited number of alloherpesviruses have been fully sequenced and characterised to date [7]. This scarcity is likely attributable to the high genomic complexity and diversity of these viruses, as well as the historical lack of research focus on aquatic organisms. Consequently, this study provides a timely contribution to our understanding of alloherpesvirus genomics and evolution, thereby facilitating future comparative research and the discovery of related viruses in both pike and other species.

## Data Availability

Information about sample and raw sequencing data are available at the National Center for Biotechnology Information (NCBI) under the BioProject PRJNA1191668 and the annotated EsHV-1 genome sequence are given accession number PV991061.1.

## Supplementary Materials

The following supporting information can be downloaded at: https://www.mdpi.com/article/10.3390/v17101361/s1, Figure S1: IRS dot-plots; Figure S2: ORF1, 38, 63 dot-plots; Figure S3: Neighbour-joining tree of ten concatenated alloherpesvirus core proteins.

## Abbreviations

The following abbreviations are used in this manuscript:

AciHV-2	Ictavirus acipenseridallo2
CDS	Coding sequence
EsHV-1	Esocid Herpesvirus 1
IRS	Inverted repeat sequence
NGS	Next-generation sequencing
ORF	Open reading frame
SalHV-1	Salmovirus salmonidallo1
UL	Unique long segment
US	Unique short segment
WSHV-1	White sturgeon herpesvirus 1
