# Supplementary material for: Genome Characterisation of Esocid Herpesvirus 1 (EsHV-1)
Source: Viruses. 2025 Oct 11;17(10):1361. doi: 10.3390/v17101361 (PMC12567609; doi:10.3390/v17101361)
Supplement: Supplementary file 1 [file viruses-17-01361-s001.zip › viruses-3895420-supplementary.pdf]

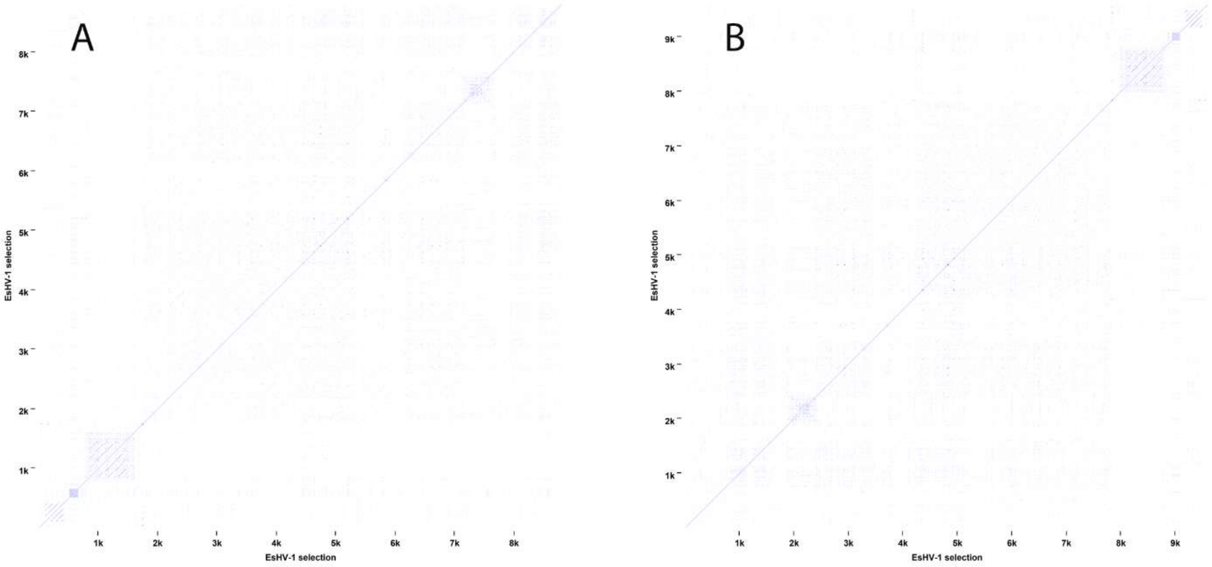

Figure S1. Dot-plot showing the IRS region between bp 154269-162989 (A) and between bp 207994-217444 (B). The similarity cutoff level is set to 70%.

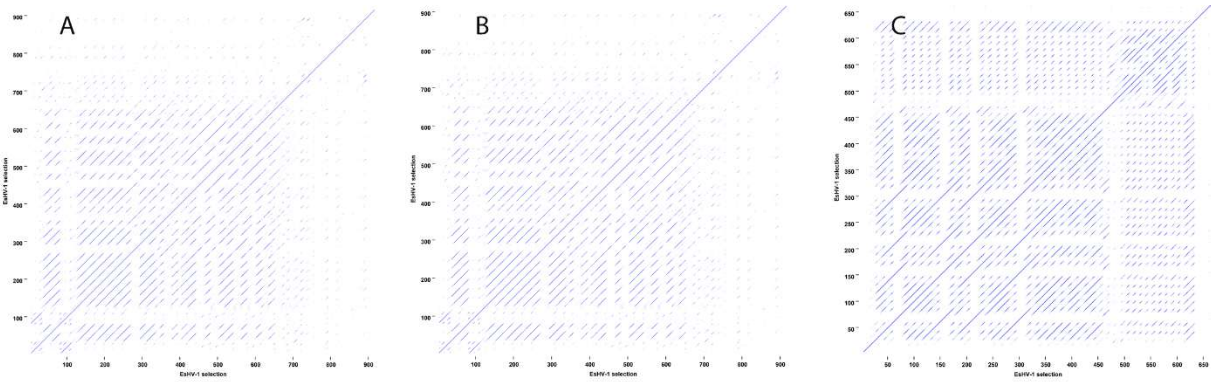

Figure S2. Dot-plot showing the ORF1 region between bp 1-903 (A), the ORF38 region between bp 48264-49864 (B) and the ORF63 region between bp 93120-93780 (C). The similarity cutoff level is set to 70%.

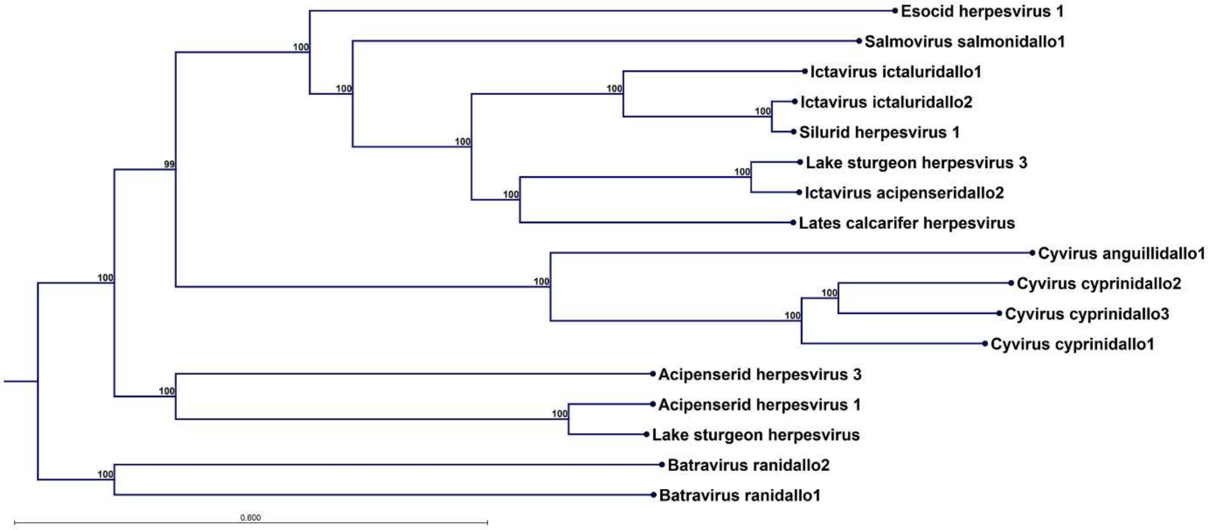

Figure S3. Neighbour-joining tree constructed from concatenated alignments of the 10 alloherpesvirus core proteins: helicase-primase subunits (helicase and primase), major capsid protein, capsid triplex subunit 2, capsid maturation protein, and the Allo37, Allo54, Allo56, Allo60, and Allo64 proteins. The bootstrap support, from 1000 replicates, is shown at the nodes. The bar signifies the branch length corresponding to the given number of substitutions per site.
